# The Role of Oxidative Stress and the Potential Therapeutic Benefits of *Aronia melanocarpa* Supplementation in Obstructive Sleep Apnea Syndrome: A Comprehensive Literature Review

**DOI:** 10.3390/antiox13111300

**Published:** 2024-10-26

**Authors:** Alicja Jelska, Agnieszka Polecka, Andrii Zahorodnii, Ewa Olszewska

**Affiliations:** 1 Doctoral School, Medical University of Bialystok, 15-089 Bialystok, Poland; agnieszka.polecka@sd.umb.edu.pl (A.P.);; 2 Department of Otolaryngology, Sleep Apnea Surgery Center, Medical University of Bialystok, 15-089 Bialystok, Poland

**Keywords:** sleep apnea, obstructive sleep apnea, oxidative stress, biomarkers, inflammation, *Aronia melanocarpa*, black chokeberry, antioxidant

## Abstract

Obstructive sleep apnea (OSA) is a sleep disorder characterized by repeated episodes of apnea and hypopnea, leading to inflammation and oxidative stress that contribute to numerous health complications, including cardiovascular diseases. Continuous positive airway pressure (CPAP) is a standard for treating OSA and is effective in reducing inflammation and oxidative stress. *Aronia melanocarpa* (a black chokeberry), a deciduous shrub belonging to the Rosaceae family and native to eastern North America that is rich in polyphenols, has garnered attention for its therapeutic potential due to its ability to neutralize reactive oxygen species (ROS) and modulate inflammatory pathways, such as NF-κB. This review supports the hypothesis that combining CPAP with black chokeberry supplementation might provide a more comprehensive approach to treating OSA, reducing the risk of health complications by further reducing oxidative stress. In conclusion, *Aronia melanocarpa* has potential benefits as an adjunct therapy in the treatment of OSA, enhancing overall health and well-being. This review compiles the latest scientific findings on the benefits of black chokeberry supplementation, its application in OSA treatment, and its potential use in the treatment of other conditions linked to oxidative stress.

## 1. Introduction

Obstructive sleep apnea (OSA) is the most common sleep-related respiratory disorder. It is characterized by repeated episodes of apnea, which are the result of a weakening of the muscles responsible for the expansion of the upper respiratory tract during sleep, leading to hypoxia, later hypoxemia, and consequently hypercapnia. Depending on the apnea/hypopnea index (AHI), OSA can be divided into mild (5 ≤ AHI < 15), moderate (15 ≤ AHI < 30) and severe (AHI ≥ 30). The prevalence of OSA is estimated at approximately 1 billion adults aged 30–69 years [1]. The risk factors for obstructive sleep apnea include male gender, older age, ethnicity, family history, increased body mass index (BMI), obesity (including central body fat distribution and large neck circumference), craniofacial and upper airway abnormalities, and detrimental behavioral patterns, e.g., alcohol consumption and smoking [2,3,4]. Untreated OSA is associated with a higher risk of morbidity and mortality due to cardiovascular diseases, heart failure, stroke, pulmonary hypertension, metabolic disorders, diabetes mellitus, neurological diseases, depression, and cognitive disorders [5,6,7]. Additionally, recent scientific reports indicate that OSA plays an important role as a pathogenetic factor in insulin resistance, hypertension, cancer, diseases of the immune system, kidney diseases, and non-alcoholic fatty liver disease (NAFLD) as well as affecting the hearing system at multiple levels [8,9,10,11]. 

The standard treatment for OSA involves continuous positive airway pressure (CPAP) therapy, which maintains airway patency during sleep [12]. While CPAP is highly effective, adherence remains a significant challenge for many patients due to discomfort or inconvenience [13]. Additionally, CPAP primarily addresses the mechanical aspects of airway obstruction but does not fully mitigate the underlying oxidative stress and systemic inflammation caused by recurrent hypoxic episodes. The oxidative stress resulting from OSA-induced hypoxia–reoxygenation cycles is a key contributor to cardiovascular and metabolic disorders. This pathophysiological process generates an excess of reactive oxygen species (ROS), overwhelming the body’s natural antioxidant defenses and leading to endothelial dysfunction, inflammation, and a heightened risk of cardiovascular events [14]. Current treatments for OSA do not adequately address this oxidative burden, suggesting the need for adjunctive therapies that specifically target the inflammatory and oxidative pathways involved in OSA progression.

*Aronia melanocarpa*, frequently referred to as the black chokeberry, is native to North America and belongs to the Rosaceae family, which includes many plants of significant economic and ecological importance [15]. This family comprises a wide range of fruits, for instance, apples, pears, plums, and peaches, as well as various shrubs and trees. Black chokeberry fruits are one of the most potent natural antioxidants [16]. *Aronia* has been highlighted for its potent antioxidative and anti-inflammatory properties due to its high concentration of polyphenolic compounds, including phenolic acids (chlorogenic and neochlorogenic), and flavonoids, e.g., anthocyanins, proanthocyanidins, flavanols, and flavonols. The characteristic dark purple color of Aronia fruits is due to the presence of anthocyanins, which are concentrated in the outer part of the fruit skin. Proanthocyanidins are responsible for the pungent taste of fruit, which can be a problem in consumption. Consequently, Aronia is often processed into products such as wines, juices, teas, preserves, jams, granola bars, and encapsulated extracts to enhance its consumption. The flavonoid group, which is most prevalent in Aronia fruits, includes cyanidin-3-O-galactoside, cyanidin-3-O-arabinoside, epicatechin, and quercetin [17,18,19,20,21]. These compounds have demonstrated the ability to neutralize free radicals, reduce oxidative stress, and improve endothelial function in various clinical settings, including hypertension and metabolic syndrome, common comorbidities in OSA [22]. Moreover, chokeberry supplementation presents a promising adjunctive approach in OSA management by addressing the oxidative and inflammatory components. In this context, chokeberry could play a dual role: supporting cardiovascular health in OSA patients as well as potentially improving the outcomes of standard treatments by mitigating oxidative damage and systemic inflammation. Integrating antioxidative supplements like *Aronia melanocarpa* into the treatment paradigm may represent a novel and complementary strategy to enhance the overall management of OSA, particularly for patients who experience suboptimal benefits from CPAP therapy alone. Thus, this review explores the potential of chokeberry supplementation as an adjunctive treatment in OSA, with a focus on its antioxidative and anti-inflammatory effects. 

## 2. Pathophysiology of OSA Biomolecular Mechanisms 

In patients with OSA, recurrent episodes of impaired airflow due to upper airway obstruction during sleep can result in apnea and hypopnea, leading to periodic fluctuations in blood oxygenation, including hypoxemia and hypercapnia. These fluctuations trigger the overproduction of reactive oxygen species as a direct consequence of intermittent hypoxemia and subsequent reoxygenation, resulting in systemic oxidative stress, and cellular damage, which are central components of OSA pathophysiology [23,24,25]. Systemic oxidative stress in OSA might represent a key mechanism of underlying endothelial dysfunction and is a major contributor to the increased cardiovascular risk disease observed in these patients [26].

Reactive oxygen species, such as superoxide anions, hydroxyl radicals, and hydrogen peroxide, are highly reactive molecules that damage cellular components like lipids, proteins, and DNA. In conditions like OSA, repeated cycles of hypoxia and reoxygenation lead to excessive ROS production, overwhelming the body’s natural antioxidant defences [27]. This results in oxidative stress, contributing to tissue damage, inflammation, and the progression of comorbid conditions like cardiovascular disease.

Furthermore, the presence and excess of ROS diminish the synthesis of nitric oxide (NO), a critical molecule for vascular relaxation, vascular tone regulation, and immune responses. Increased ROS levels and NO depletion synergistically contribute to vascular inflammation, and endothelial cell apoptosis, thus influencing multiple components of cellular signaling pathways, such as IκB kinase (IKK), which phosphorylates the IκB protein, leading to its degradation and the subsequent release of NF-κB. Once activated, NF-κB translocases to the nucleus, where it initiates the transcription of genes encoding for proteins involved in the inflammatory response, including pro-inflammatory cytokines (TNF-α, IL-6, IL-1β, and IL-10) and adhesion proteins [25,28,29,30,31]. The NF-κB pathway is pivotal in the chronic inflammation observed in OSA, exacerbating oxidative stress and contributing to a vicious cycle of cellular damage and chronic inflammation by the generation of ROS [32]. This ongoing inflammatory response not only exacerbates existing cellular injury but also amplifies oxidative stress, further degrading cellular integrity and impairing the function of various biological systems [33]. Additionally, oxidative stress impairs vascular endothelial function by reducing NO bioavailability, contributing to the development of cardiovascular complications [34]. This dysfunction is characterized by increased vascular permeability, leukocyte adhesion, and atherosclerotic plaque formation, all of which contribute to the cardiovascular complications associated with OSA [35]. These mechanisms underscore the crucial role of oxidative stress in the pathophysiology of OSA, affecting multiple biological systems and thus leading to significant health complications. 

## 3. Underlying Mechanisms of *Aronia melanocarpa* Antioxidant Properties and Their Role in Oxidative Stress 

The antioxidant properties of black chokeberry fruits are highly dependent on the presence of polyphenolic compounds, which are responsible for their diverse mechanisms of action. Polyphenols, including chlorogenic acid, epicatechin, quercetin, and anthocyanins, play a significant role in scavenging free radicals and protecting cells from oxidative damage [36]. Moreover, polyphenols donate electrons to ROS, effectively neutralizing them before they cause significant damage [37]. In addition to their antioxidant activity, polyphenolic compounds in black chokeberry are also responsible for inhibiting 15-lipoxygenase (15-LOX) and xanthine oxidase, which are both involved in the generation of ROS [38]. Inhibition of 15-LOX can reduce tissue injury by suppressing the production of pro-inflammatory cytokines and leukotrienes, infarct size, and lipid peroxidation, potentially mitigating inflammatory responses associated with various chronic conditions [39]. Similarly, the inhibition of xanthine oxidase decreases the formation of uric acid and superoxide radicals, providing further protection against ROS production, and oxidative stress, which contribute to the morbidity associated with OSA [25].

One of the most significant capabilities of Aronia fruit is the ability to neutralize radicals, which is strongly correlated with the overall polyphenol content. Anthocyanins, the pigments responsible for Aronia’s dark color, are particularly effective due to their unique structure, which allows them to stabilize free radicals by the electron donation process [40]. It prevents ROS from interacting with key cellular components, such as lipids and DNA. In comparison to other fruits and berries, Aronia also demonstrates the highest activity in scavenging free radicals as evidenced by the oxygen radical absorbance capacity (ORAC) test [41]. The ORAC test is a laboratory method that quantifies the antioxidant capacity of a sample to neutralize free radicals. A higher ORAC value indicates a greater antioxidant capacity, meaning that the substance is more effective in safeguarding the body from oxidative stress. The exceptional ORAC level of Aronia not only highlights its potential as a dietary antioxidant but also suggests its efficacy in the prevention of chronic diseases. This positions Aronia as an exceptionally potent component in nutraceuticals and superfoods, aimed at enhancing overall health and well-being, further emphasizing its value as a natural remedy for combating oxidative stress.

## 4. Importance of Oxidative Stress Biomarkers and *Aronia melanocarpa* in OSAS Pathophysiology

Oxidative stress is associated with several beneficial biomarkers that could prove valuable for the future in OSA diagnostics. These include 8-isoprostanes, 8-hydroxydeoxyguanosine (8-OHdG), superoxide dismutase (SOD), malondialdehyde (MDA), total oxidative status (TOS), total antioxidant status (TAS), and total antioxidant capacity (TAC). These markers provide critical information on the extent of oxidative damage and the body’s antioxidant defense, which are significantly compromised in OSA due to ongoing pathophysiological processes. 

8-isoprostane is a byproduct of lipid peroxidation and is widely regarded as a sensitive biomarker of oxidative stress [42]. ROS targets cellular membranes, leading to lipid peroxidation—a process where ROS oxidizes the lipids in cell membranes, causing structural damage and cell dysfunction [43]. Elevated 8-isoprostane levels reflect high oxidative damage to lipids, which is commonly seen in OSA patients. Polyphenols in Aronia, especially proanthocyanidins, inhibit the lipid peroxidation process by preventing ROS from attacking the lipids in cell membranes. These polyphenols have strong hydrogen-donating abilities and can chelate metal ions (iron (Fe^2+^) and copper (Cu^2+^)) that catalyze free radical formation. This reduces the propagation of lipid peroxidation chains, thereby lowering 8-isoprostane concentrations and protecting the structural integrity of cell membranes. As part of the existing literature, a study conducted by Villa et al. involving sixty-five children with sleep-disordered breathing, with a mean age of 5.9 ± 2.0 years, reported that urinary concentration of 8-isoprostane correlated positively with clinical sleep scores and AHI, athough negatively with age and body surface area. Notably, urinary 8-isoprostane concentration was significantly higher in the group with AHI ≥5 compared to the group with the AHI group < 5 (*p* < 0.01) [44]. Similarly, a study conducted by Petrosyan et al. demonstrated that the AHI was positively correlated with 8-isoprostane concentrations in exhaled air, further implicating oxidative stress in the pathophysiology of OSA [45]. These findings highlight the potential of 8-isoprostane as a valuable biomarker for monitoring disease severity and progression. It also suggests a promising avenue for future research, focused on targeted interventions to mitigate oxidative stress and improve clinical outcomes in OSA patients. 

Patients with OSA also exhibit elevated concentrations of 8-hydroxydeoxyguanosine (8-OHdG), a marker of DNA damage induced by oxidative stress. Elevated concentrations of 8-OHdG are not only indicative of DNA oxidation but are also associated with mutations and carcinogenesis, especially in diseases linked to chronic oxidative stress, such as OSA. A study by Gille et al. analyzed the 8-OHdG concentration in patients with recently diagnosed idiopathic pulmonary fibrosis, stratified by OSA severity. The results showed significantly higher 8-OHdG concentrations in patients with severe OSA compared to those with mild or moderate OSA, suggesting that severe OSA is associated with increased oxidative stress, as evidenced by elevated levels of this DNA damage marker [46]. Aronia’s antioxidant capacity helps protect DNA integrity by reducing the accumulation of ROS that can attack nucleotides. 

Flavonoids in Aronia have been shown to bind directly to DNA and might protect against ROS-induced strand breaks by either scavenging the free radicals before they reach the DNA or stabilizing the DNA structure, making it less susceptible to oxidative attack, which renders them potent antioxidants [47,48]. Furthermore, the polyphenol component of black chokeberry fruits increases the expression and activity of antioxidant enzymes such as superoxide dismutase, catalase and glutathione peroxidase, and also reduces the activity of ROS-producing enzymes such as NADPH oxidase [49]. By lowering ROS levels, Aronia indirectly reduces the activation of pro-inflammatory pathways, such as NF-κB, which is often triggered by oxidative stress and is involved in the body’s inflammatory response. This protective effect on DNA integrity could help mitigate the long-term consequences of oxidative stress, such as genomic instability, premature aging, and the development of cancer. 

Superoxide dismutase (SOD), a critical antioxidant enzyme involved in the detoxification of superoxide radicals, is another important indicator for monitoring the antioxidant status in OSA. It is responsible for defending against oxidative damage by catalyzing the dismutation of the superoxide anion (O_2^−^_), a highly reactive ROS, into less harmful molecules—hydrogen peroxide (H_2_O_2_) and molecular oxygen (O_2_). In conditions like obstructive sleep apnea (OSA), repeated hypoxia–reoxygenation cycles lead to the overproduction of superoxide anions, overwhelming the body’s natural SOD reserves and resulting in oxidative stress. A meta-analysis coordinated by Pau et al. revealed that the SOD concentration in whole blood samples was significantly lower in patients with OSA compared to the control group. This finding suggests impaired antioxidant defense in OSA and provides new directions for further studies [50]. Aronia’s polyphenolic components, particularly anthocyanins, and flavonoids, not only act as direct scavengers of ROS but also stimulate the expression and activity of SOD. By upregulating SOD activity, Aronia helps the body more efficiently detoxify superoxide anions, reducing oxidative damage at its source. This enhanced antioxidant defense lowers the risk of oxidative injury to critical cellular components such as DNA, lipids, and proteins. Furthermore, improved SOD activity has downstream effects, reducing the overall oxidative burden and minimizing inflammation, which is critical in OSA-associated cardiovascular and metabolic diseases.

Malondialdehyde (MDA) is a highly reactive byproduct of lipid peroxidation, where ROS, particularly superoxide and hydroxyl radicals, attack polyunsaturated fatty acids in cellular membranes. Elevated MDA levels are a hallmark of increased oxidative stress and cellular membrane damage, making it a key biomarker in conditions like OSA, where oxidative stress is pervasive due to intermittent hypoxia [51]. Another meta-analysis conducted by Pau et al. confirmed that the MDA concentration was significantly higher in patients with OSA than in controls, reinforcing the role of oxidative stress in the pathogenesis of OSA [52]. Aronia’s antioxidants help protect lipids from peroxidation through both direct and indirect mechanisms. The polyphenols in Aronia, especially proanthocyanidins, neutralize ROS before they can initiate lipid peroxidation. Additionally, Aronia’s polyphenols have been shown to chelate metal ions (such as Fe^2+^ and copper Cu^2+^) that catalyze the formation of ROS via the Fenton reaction, further reducing the oxidative processes that lead to MDA formation [53]. By lowering MDA levels, Aronia supplementation preserves the structural integrity of cell membranes, preventing functional loss and damage to vital tissues, particularly in vascular and respiratory systems commonly affected in OSA.

Total antioxidant capacity (TAC) and total antioxidant status (TAS) are comprehensive measures of the body’s ability to counteract oxidative stress. TAC is a comprehensive measure of the cumulative action of all antioxidants present in plasma and body fluids, including enzymatic antioxidants like SOD, catalase, and glutathione peroxidase, as well as non-enzymatic antioxidants like vitamins C and E, uric acid, and polyphenolic compounds derived from the diet. TAS assesses the overall antioxidant status of a sample by measuring the activity of all present antioxidants (enzymes, vitamins, and other compounds), thus measuring the net effect of all antioxidants in neutralizing free radicals. In a 2022 study by Venza et al., individuals with obstructive sleep apnea syndrome exhibited decreased TAC concentrations in saliva samples, indicating an imbalance in oxidative–reduction processes [54]. *Aronia melanocarpa* has been shown to significantly increase TAC due to its rich polyphenolic profile. Anthocyanins, flavonoids, and proanthocyanidins in Aronia act synergistically with the body’s endogenous antioxidant systems, enhancing both the scavenging of free radicals and the regeneration of other antioxidants, such as vitamin C. This dual mechanism amplifies the body’s overall antioxidant capacity, improving its resilience to oxidative stress. Additionally, TAC is associated with lower levels of oxidative stress biomarkers, such as MDA and 8-OHdG, reducing the risk of oxidative injury to vital organs and systems, particularly the cardiovascular system, which is highly vulnerable in OSA [55,56].

Total Oxidant Status (TOS) is a biomarker that quantifies the overall oxidative burden by measuring the cumulative concentration of oxidative agents in plasma providing critical insights into oxidative stress. In OSA, TOS is often elevated due to the excess generation of ROS from repeated hypoxia–reoxygenation cycles. Research performed by Olszewska et al. demonstrated that TAS was lower in the uvular mucosa of OSA patients, while TOS and oxidative stress index (OSI) were elevated, further supporting the role of oxidative stress in the pathophysiology of OSA [57]. It indicates that evaluating these biomarkers could be instrumental in tracking the disease progression and assessing the effectiveness of therapeutic interventions. Aronia’s polyphenolic compounds help reduce TOS by directly neutralizing ROS and inhibiting their production. The ability of Aronia’s antioxidants to quench free radicals not only lowers immediate oxidative damage but also reduces the activation of pro-inflammatory pathways, such as NF-κB, which are often triggered by oxidative stress. This reduction in oxidative and inflammatory signaling helps lower the TOS, reflecting a decrease in the overall oxidative burden. A lower TOS is critical for preventing long-term complications, such as endothelial dysfunction, atherosclerosis, and metabolic disturbances, all of which are linked to oxidative stress in OSA. Additional indicators, including advanced glycation end products (AGEs), and thiobarbituric acid reactive substances (TBARSs), offer substantial insights into the oxidative state and potential cellular injury associated with OSA [58,59,60].

Monitoring these biomarkers (Table 1) can provide valuable information on disease severity and therapeutic efficacy, enabling more precise treatment adjustments. Integrating data from these markers opens new avenues for understanding the underlying mechanisms of OSA, supporting the development of more effective therapeutic strategies.

## 5. The Effects of OSA Treatment on Oxidative Stress Levels

Continuous positive airway pressure (CPAP) is one of the non-invasive methods, which is considered the gold standard of treatment for OSA. It can be delivered through a variety of devices designed to maintain the patency of the upper airways during sleep. This therapy effectively reduces the incidence of apneas, hypopneas, intermittent hypoxia, reoxygenation, and nocturnal arousals. Additionally, CPAP has been shown to reduce oxidative stress, primarily by stabilizing oxygen levels during sleep, thereby reducing the frequency and severity of hypoxic episodes [61,62]. This stabilization limits the overproduction of reactive oxygen species, which are known contributors to oxidative damage and inflammation. Karamani et al. demonstrated that a three-month course of CPAP treatment resulted in a substantial decrease in serum concentrations of 8-isoprostane [61]. Similarly, a study conducted by Jurado-Gámez et al. revealed that eight weeks of CPAP therapy significantly decreased the concentration of 8-OHdG (8-hydroxy-2′-deoxyguanosine) [63]. In contrast, Borges et al. reported that after 8 weeks of either CPAP or aerobic exercises, there were no significant differences in the concentration of cell-free DNA, oxidative stress, and inflammation markers, including advanced oxidation protein products (AOPPs) and superoxide dismutase (SOD). However, there was a notable improvement in both sleep efficiency and the duration of sleep [64]. CPAP therapy not only enhances the quality of sleep but also decreases the risk of cardiovascular diseases and other conditions associated with chronic inflammation. These findings underscore the importance of CPAP as a relevant intervention not only in managing the symptoms of OSA but also in reducing systemic oxidative stress and its associated complications. 

### Oxidative Stress in OSA and CPAP Therapy: Aronia melanocarpa, a Missed Opportunity?

**Figure 1:**
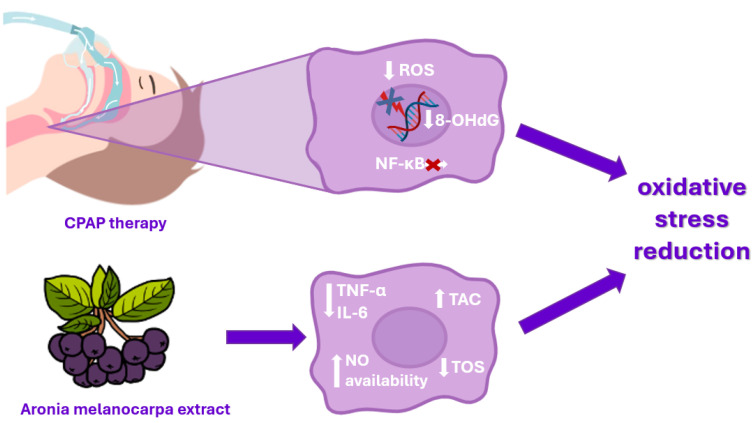
Effects of combination CPAP therapy and Aronia melanocarpa extract supplementation on oxidative stress reduction.

The damage caused by oxidative stress in OSA patients is not limited to the airway; it extends to vital organs, including the cardiovascular system. OSA is strongly associated with hypertension, atherosclerosis, and other cardiovascular diseases due to this chronic oxidative damage. CPAP therapy significantly improves oxygenation and reduces hypoxic episodes. However, CPAP does not directly address the oxidative stress already present in the body, nor does it reverse the oxidative damage that has accumulated over time. Furthermore, some patients may continue to experience residual oxidative stress even with effective CPAP therapy. This is where *Aronia melanocarpa* supplementation could potentially enhance patient outcomes by targeting and reducing oxidative stress more directly. The synergistic effects of combining *Aronia melanocarpa* supplementation with CPAP therapy in the management of OSA are illustrated in Figure 1. Firstly, Aronia can lower concentrations of pro-inflammatory cytokines, such as TNF-α and IL-6, both of which are elevated in OSA patients [65]. This reduction in inflammation could improve cardiovascular outcomes and reduce the risk of long-term complications. Secondly, polyphenols, particularly anthocyanins, have been shown to improve endothelial function by increasing NO availability, which helps regulate vascular tone and reduce blood pressure. Improved endothelial function could decrease the risk of atherosclerosis and other cardiovascular complications in OSA patients. Thirdly, polyphenolic compounds contribute significantly to TAC by providing a high concentration of antioxidants that synergize with the body’s endogenous defense systems. By increasing TAC, Aronia supplementation can help OSA patients achieve a better oxidative balance, reducing oxidative stress markers such as MDA and 8-OHdG. Additionally, by reducing TOS, Aronia helps shift the oxidative balance in favor of antioxidant protection, improving overall health outcomes.

## 6. Potential Therapeutic Effects of *Aronia melanocarpa* in Other Conditions

Recently, *Aronia melanocarpa* has gained the attention of many scientists due to its rich profile of health-promoting substances including polyphenols, anthocyanins, and other vitamins or minerals. These natural components of the plant increased the potential of future research. The diverse polyphenol profile and natural substances contained in Aronia, combined with its high antioxidant capacity, position it as a promising natural remedy with broad applications in promoting general well-being, preventing various chronic diseases and reducing oxidative stress [66,67]. The mechanisms of action of black chokeberry consist of inhibiting the NF-κB pathway, leading to the inhibition of ROS production [68,69]. It has also been shown that the extract of these fruits contributes to the increase in NO production [70], improving vascular mobility.

### 6.1. Metabolic Effects of Aronia melanocarpa

Recent research has highlighted the significant potential of black chokeberry in improving lipid profiles, particularly in lowering low-density lipoprotein (LDL) and total cholesterol levels [71]. Its lipid-modulating effects are crucial for reducing the risk of cardiovascular disease. Notably, a study by Christiansen et al. demonstrated that only long-term supplementation with *Aronia* (6 weeks to 3 months) effectively decreased LDL cholesterol, whereas shorter treatment durations did not yield the same effect [72]. Similarly, Daskalova et al. found that three months of supplementation with functional beverages derived from black chokeberry resulted in a statistically significant increase in HDL concentration in aging rats, while concurrently reducing markers indicative of atherosclerosis (LDL, triglycerides). This suggests a beneficial impact of Aronia on lipid profile and cardiovascular health [73]. In another study by Tasic et al., patients were divided into groups based on gender and the presence of metabolic syndrome or both metabolic syndrome and type 2 diabetes mellitus. The study revealed that after 4 weeks of administration of a standardized preparation of Aronia extract (SAE) at a dose of 400 mg, total cholesterol and LDL concentrations significantly decreased in both groups compared to the baseline. Triglycerides were significantly reduced only in diabetic individuals, while HDL concentration increased in women with metabolic syndrome [74]. Although Aronia shows promise in increasing HDL concentration, further research is necessary to confirm these effects in healthy individuals.

Chokeberries might also positively influence glucose metabolism. The study conducted by Christiansen C. B. et al. reported a significant reduction in blood glucose concentration by 7.92 mg/dL in patients receiving Aronia supplementation compared to control groups [72]. Furthermore, a prospective controlled trial discovered that individuals with metabolic syndrome or type 2 diabetes mellitus experienced improved glycemic control following four weeks of Aronia extract administration (400 mg of polyphenols) [74]. The mechanisms underlying these effects were further elucidated by Chen et al., who found that anthocyanin extracts from *Aronia melanocarpa* enhance glucose uptake by upregulating glucose transporter-4, potentially improving insulin sensitivity in HepG2 (human hepatocellular carcinoma) and C2C12 (mouse myogenic) cells. This upregulation increases insulin receptor substrate-1 (IRS-1) levels while decreasing phosphorylated IRS-1 (p-IRS-1) levels, thereby promoting glycogen synthesis [75].

### 6.2. Cardiovascular Benefits of Aronia melanocarpa

The development of hypertension is primarily attributed to endothelial dysfunction, where the inner lining of blood vessels fails to regulate vascular tone effectively. Numerous studies have demonstrated that this process might be associated with oxidative stress, characterized by the overproduction of ROS. This oxidative imbalance can lead to alterations in TAC levels, contributing to the development of various cardiovascular diseases [76]. Free radicals damage the endothelium, leading to vasoconstriction, inflammation, and ultimately, persistent elevation of blood pressure. Recent studies have indicated that daily consumption of chokeberries can help regulate blood pressure by reducing oxidative stress and enhancing endothelial function, thus offering protection against hypertension [77]. The antihypertensive effect of chokeberries is further enhanced and by the reduction in endothelin-1, a potent vasoconstrictor, and by boosting the activity of endothelial NO synthase [78,79]. A meta-analysis conducted by Hawkins et al. revealed that daily supplementation with Aronia berry, which was an active component in extracts, capsules, or beverage products for 6–8 weeks, significantly reduces systolic blood pressure [80]. These findings suggest potential therapeutic applications for *Aronia* in the management of hypertension, warranting further investigation in future research. This effect is particularly important in patients with coexisting OSA, as it is one of the key factors contributing to the development of hypertension in this population. The prevalence of hypertension in patients with OSA ranges from 35% to 80% and may be closely related to the severity of the condition [81].

### 6.3. The Role of Aronia melanocarpa Polyphenols in Gut Health and Prebiotic Potential

In the context of digestive health, polyphenols are the most valuable components of *Aronia melanocarpa*. According to research, their consumption can stimulate the production of short-chain fatty acids, e.g., butyrate and propionate that support the integrity of the intestinal barrier and protect endothelial cells from oxidative stress-induced damage [82]. Additional studies by Le Sayec et al. demonstrated that the consumption of polyphenols from black chokeberry significantly increased both the gene richness of the gut microbiome and the number of butyrate-producing species, including Intestinimonas butyriciproducens and Lawsonibacter asaccharolyticus. These results emphasize the close relationship between vascular elasticity, gut microbiome health, and phenolic compounds derived from black chokeberry, as confirmed by metabolomic, metagenomic, and clinical studies [83]. Furthermore, *Aronia melanocarpa* was identified as a potential prebiotic strategy for treating obesity-induced colonic inflammation [84]. 

### 6.4. Immunomodulating Effects of Aronia melanocarpa

The fruit of *Aronia melanocarpa* is abundant in polyphenols and other bioactive compounds, which can enhance the defense mechanisms by influencing both innate and adaptive immunity. Its immunomodulatory features are particularly beneficial in managing chronic inflammation and supporting overall immune function. In a study conducted by Badescu et al., it was discovered that isolated polyphenols from black chokeberry modulated both types of response-targeted and non-targeted in individuals with insulin-deficient diabetes, decreasing the inflammatory condition and self-sustaining pancreatic insulitis [85]. Another study performed by Bushmeleva et al. indicated that black chokeberry promotes the rapid recuperation of the immune system in rats, normalizes the leukocyte count, and enhances the phagocytic activity of monocytes and neutrophils [86]. The special immunomodulatory effect of the fruit is attributed to the compounds, such as cyanidin-3-O-galactoside, and cyanidin-3-O-glucoside, which reduce oxidative stress and modulate cytokine production [87]. These processes play a crucial role in dampening inflammatory responses and promoting immune system balance.

## 7. Discussion

The therapeutic potential of *Aronia melanocarpa* is supported by a growing body of evidence, particularly in its ability to minimize oxidative stress, inflammation, and endothelial dysfunction, which are crucial in OSA pathogenesis. The polyphenols contained in black chokeberry fruit, including anthocyanins, quercetin, and epicatechin, exhibit significant antioxidant and anti-inflammatory effects [36,88], which are key in mitigating molecular mechanisms that contribute to the onset and progression of OSA. The proven strong antioxidant properties of *Aronia* may help reduce ROS levels and improve endothelial function, potentially reducing the risk of cardiovascular disorders in patients with OSA [70,89]. Equally important and promising is the synergistic potential of *Aronia melanocarpa* in conjunction with CPAP therapy. CPAP therapy effectively stabilizes oxygen levels and reduces intermittent hypoxia [90], while *Aronia melanocarpa* might enhance these effects by decreasing markers of oxidative stress [91], including 8-isoprostanes and 8-OHdG, which are commonly elevated in OSA patients. Furthermore, by inhibiting pro-inflammatory pathways and modulating NF-κB signaling pathway, Aronia may help reduce systemic inflammation and improve immunomodulation [87,92]. This reduction in chronic inflammation could increase the efficacy of CPAP therapy and improve patient outcomes. 

In addition, there is growing interest in pharmacological treatments for OSA. Recent research has highlighted the potential of therapeutic agents, such as Tirzepatide, which is currently undergoing phase III of clinical trials for OSA [93]. Other pharmacological possibilities include a combination of Atomoxetine with Aroxybutynin/Oxybutynin, which resulted in a decreasing apnea–hypopnea index [94,95]. This opens up opportunities for new directions of research into the pharmacological treatment of OSA.

Moreover, it is important to note that effective doses of *Aronia melanocarpa* can vary significantly between studies. Currently, there is no established standardized dose, and further research is required to determine the optimal dosage, form, and duration for therapeutic use. The variability in *Aronia*’s effects may be due to differences in polyphenol concentrations, which are influenced by factors such as processing methods, and bioavailability [72,96]. Therefore, future studies must explore and test a range of dosages to identify what is most effective in clinical settings.

Future studies should aim to elucidate the specific molecular mechanisms by which *Aronia melanocarpa* influences key enzymes involved in ROS production, such as NADPH oxidase and xanthine oxidase, as well as its role in modulating NO availability and endothelial nitric oxide synthase (eNOS) activity. Investigation into these pathways may provide valuable insights into how Aronia improves vascular health and ameliorates mitigates endothelial dysfunction, which is crucial for preventing cardiovascular complications frequently associated with OSA.

Additionally, long-term clinical trials are needed to determine the optimal dose, form and duration of *Aronia melanocarpa* use in patients with OSA. These studies should focus on evaluating the effects of black chokeberry on specific biomarkers of oxidative stress, inflammation, and endothelial function, as well as its effects on the incidence of cardiovascular events and metabolic disorders. Investigating Aronia’s potential to reduce the severity of OSA symptoms, such as daytime sleepiness and fatigue, is also important, as this could further improve patient compliance and quality of life. 

## 8. Conclusions and Future Directions

Beyond its application in OSA, the findings on *Aronia melanocarpa* hold broader implications for the treatment of other conditions characterized by oxidative stress and inflammation, such as cardiovascular diseases, diabetes, and metabolic syndrome. The potential of *Aronia* as a natural, plant-based intervention aligns with the growing interest in nutraceuticals and functional foods as complementary therapies in chronic disease management. This positions *Aronia melanocarpa* not only as a promising adjunct in OSA treatment but also as a valuable component of integrative health strategies aimed at reducing the burden of chronic diseases associated with oxidative stress and inflammation. In conclusion, while *Aronia melanocarpa* shows considerable promise as a therapeutic adjunct in the management of OSA, further research is essential to fully elucidate its mechanisms of action and optimize its clinical application. Future research directions should include clinical trials involving human subjects to test the efficacy and safety of Aronia supplementation in OSA patients. Establishing the optimal dosage and treatment duration will be critical for its clinical application. Additionally, detailed studies exploring the molecular and physiological mechanisms by which Aronia exerts its antioxidant and anti-inflammatory effects in the context of OSA are needed to enhance our understanding and therapeutic strategies.

Integrating *Aronia* into existing treatment protocols, particularly in combination with CPAP therapy, could offer a more comprehensive approach to managing OSA and its associated complications. As research progresses, *Aronia melanocarpa* could emerge as a key player in the broader field of natural therapies, contributing to more effective and holistic treatment strategies for chronic diseases characterized by oxidative stress and inflammation.

Furthermore, it is imperative to conduct long-term clinical trials in order to determine the most effective dosage and duration of *Aronia melanocarpa* supplementation in OSA patients. These studies should primarily focus on *Aronia*’s influence on the incidence of cardiovascular events and metabolic dysregulation, as well as its effects on key biomarkers of oxidative stress, inflammation, and endothelial function. Additionally, investigating the potential of *Aronia* to reduce the severity of OSA symptoms, such as daytime sleepiness and fatigue, could further enhance patient compliance and quality of life.

## 9. Limitations

One of the primary limitations in our review is the lack of long-term clinical trials, assessing the sustained effects of *Aronia* supplementation. Currently, there is a lack of direct studies specifically investigating the impact of *Aronia melanocarpa* on OSA as well as its underlying molecular pathways. While black chokeberry has been extensively studied for its antioxidant and anti-inflammatory properties, its potential impact on the pathophysiological mechanisms of OSA remains unexplored. Additionally, the majority of studies involve clinical trials with a limited number of participants, which restricts the generalizability of their findings.

Another limitation is the insufficient understanding of the interactions, e.g., between *Aronia melanocarpa* and other therapies, including CPAP. Further research is needed to determine how *Aronia* supplementation might affect the efficacy of these supplementations or interact with other medications. While there is substantial evidence supporting the benefits of *Aronia melanocarpa*, more mechanistic studies are required to fully understand its effects on molecular pathways associated with OSA, particularly those involving oxidative stress, inflammation, and endothelial function.

## Figures and Tables

**Table 1:** Potential biomarkers for assessing oxidative stress in OSAS.

Biomarker	Description	Literature
8-isoprostanes	A marker of lipid peroxidation. Elevated levels correlate with AHI and the severity of OSA	Petrosyan, et al. (2007) [45]
8-Hydroxydeoxyguanosine (8-OHdG)	A biomarker indicating DNA damage caused by oxidative stress. Elevated levels reflect the degree of oxidative damage.	Gille et al. (2017) [46]
Superoxide Dismutase (SOD)	An enzyme that neutralizes superoxide radicals. Reduced activity indicates excessive oxidative stress.	Pau et al. (2021) [50]
Malondialdehyde (MDA)	A marker of lipid peroxidation. Increased levels correlate with the severity of OSAS and contribute to the development of atherosclerosis.	Okur et al. (2021) [51]
Total Oxidant Status (TOS)	Measures the overall level of oxidants in the blood. Elevated levels indicate oxidative–reductive imbalance.	Olszewska et al. (2021) [57]
Total Antioxidant Status (TAS)	Indicates the antioxidant capacity of the body. Reduced levels suggest oxidative–reductive imbalance.	Olszewska et al. (2021) [57]
Total Antioxidant Capacity (TAC)	Refers to the overall antioxidant capacity in the body. Low levels are associated with higher oxidative stress and severity of OSAS.	Venza, et al. (2022) [54]
Thiobarbituric Acid Reactive Substances (TBARS)	React with thiobarbituric acid, reflecting the level of lipid peroxides.	Cofta et al. (2019) [58]
Advanced Glycation End Products (AGEs)	Products formed by the non-enzymatic reaction of sugars with proteins or lipids indicate oxidative stress.	Xu et al. (2015) [59]
